# Periodic outburst floods from an ice-dammed lake in East Greenland

**DOI:** 10.1038/s41598-017-07960-9

**Published:** 2017-08-30

**Authors:** Aslak Grinsted, Christine S. Hvidberg, Néstor Campos, Dorthe Dahl-Jensen

**Affiliations:** 1 Centre for Ice and Climate, Niels Bohr Institute, University of Copenhagen, Juliane Maries Vej 30, DK-2100 Copenhagen, Denmark; 2 Research Group of High Mountain Physical Geography, Complutense University of Madrid, 28040 Madrid, Spain

## Abstract

We report evidence of four cycles of outburst floods from Catalina Lake, an ice-dammed lake in East Greenland, identified in satellite imagery between 1966–2016. The lake measures 20–25 km^2^, and lake level drops 130–150 m in each event, corresponding to a water volume of 2.6–3.4 Gt, and a release of potential energy of 10^16^ J, among the largest outburst floods reported in historical times. The drainage cycle has shortened systematically, and the lake filling rate has increased over each cycle, suggesting that the drainage pattern is changing due to climate warming with possible implications for environmental conditions in Scoresbysund fjord.

## Introduction

Catastrophic outbursts from glacier-dammed lakes represent a severe flood hazard in regions with mountain glaciers^1, 2^. Ice dammed glacial lakes form when glacier ice prevents the downward flow of freshwater as a consequence of local topography and ice dynamics. A glacial lake outburst flood (GLOF) occurs when the ice dam fails due to flotation, overflow, mechanical failure, or by ice-marginal drainage^1, 3^. The frequency of glacial lake outburst floods are expected to increase in warmer climates because of on-going glacial retreat, and may destabilize ice sheet margins^4^. Glacial lake outburst floods cause sediment transport, and have reshaped the landscape on geological time scales^5^. Only a few observations of large glacier lake outburst floods with multi-year cycles exist^6, 7^, which limits our understanding of the processes involved. In this paper, we report evidence of four cycles of outburst floods from the Catalina Lake, an ice-dammed lake in East Greenland, identified in satellite imagery between 1966–2016.

**Figure 1:**
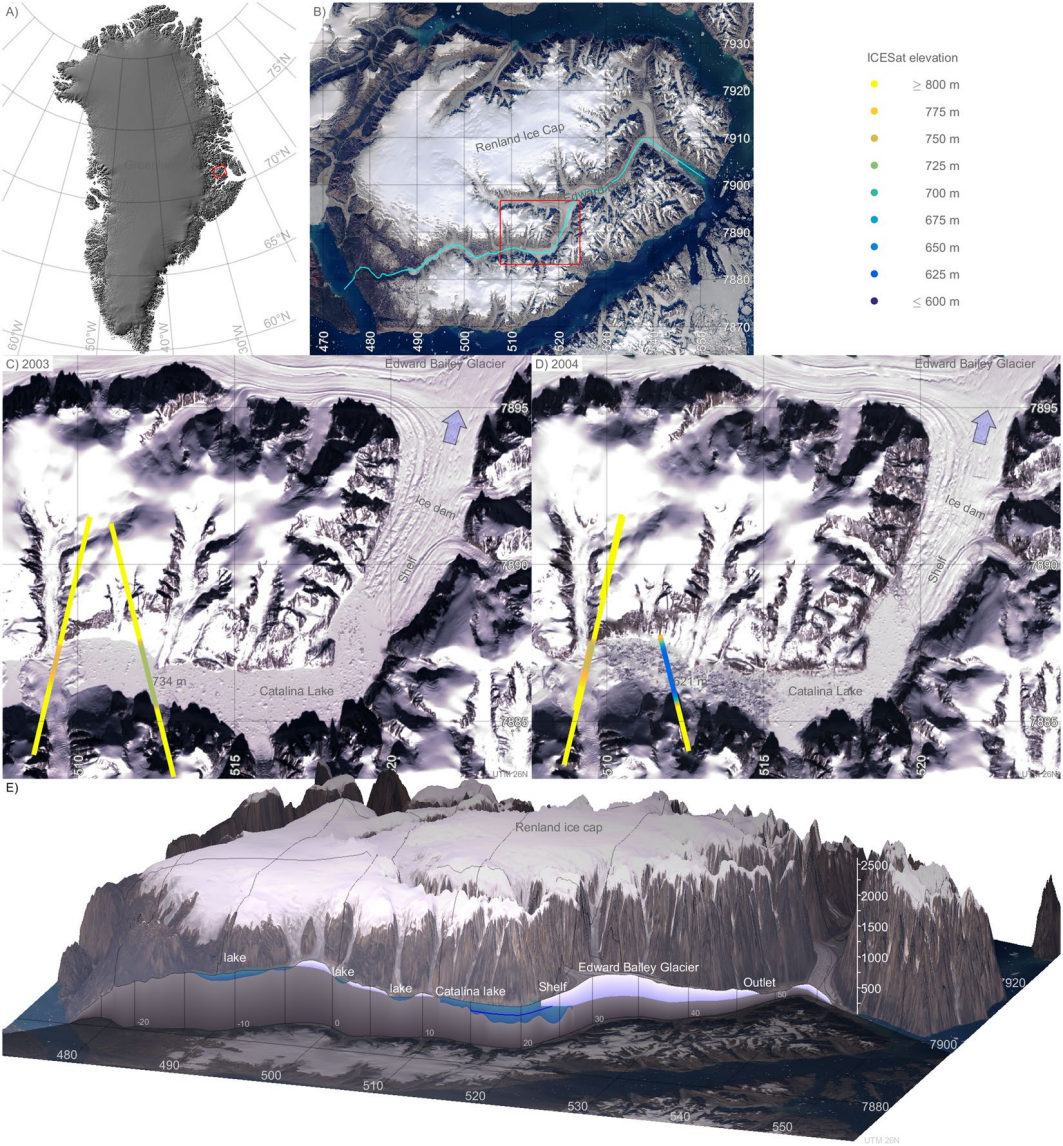
Satellite images showing the lake before and after an outburst flood. (**A**) Map of Greenland. (**B**) Map of Renland. Cyan shows location of slice in panel E. (**C**,**D**) Landsat-7 scenes showing Catalinadal valley before and after a glacial outburst flood, and elevation observations from ICESat. Light blue arrow indicate direction of drainage. (**E**) Illustrative sketch of a slice through the landscape showing location of lakes and the full 2004 lake level. The blue line shows the drained lake level in 2003. Maps were created using MATLAB^23^.

Renland is an island located in the Scoresbysund fjord, East Greenland (Fig. 1). The island is characterized by a high mountain plateau at an elevation of approximately 2 km above sea level and is surrounded by steep slopes reaching down to the fjord at sea level, typical for landscapes in East Greenland. The Renland ice cap is located on the high plateau and is drained to the south by Edward Bailey Glacier. The glacier is >40 km long and has a land-terminating tongue near sea level. At about 800 m above sea level, the glacier branches into the valley Catalinadal (71.08°N, 26.83°W) where it is fed by two smaller tributaries and calves into Catalina lake at an elevation of 600–700 m a.s.l.

Catalina Lake is the lower lake in a triple lake system, consisting of two smaller lakes at elevations ~900 m a.s.l. and ~700 m a.s.l., respectively, and Catalina Lake at ~590–730 m a.s.l. with a sub-aerial area of ~15 km^2^ (Fig. 1). Satellite observations show clear evidence of variations in Catalina Lake level over time. ICESat tracks^8^ passed over Catalina Lake in 2003 and 2004, and show a drop in lake level from 734 m a.s.l. to at least 621 m a.s.l. (Fig. 1; Supplementary dataset 1) with sub-metre accuracy^9^. The level in 2004 must be considered an upper bound as ICESat passed over a freshly exposed part of the lake bed rather than the lake (Fig. 1D). The observations indicate that a glacier lake outburst flood (GLOF) occurred between the two observations with drainage of a water volume in the order of gigatonnes from the lake system. The ICESat data constrain the timing of the drainage event to the period between November 10, 2003 and March 14, 2004. A change of the riverbed in the outwash plains downstream from the terminus of Edward Bailey Glacier after the flood is evident from Landsat images (Supplementary Figure S1), showing that the water drained via a 20 km long section of the glacier. The outburst flood from Catalina Lake is thus among the largest glacial lake drainage events recorded in historical time^1^.

**Figure 2:**
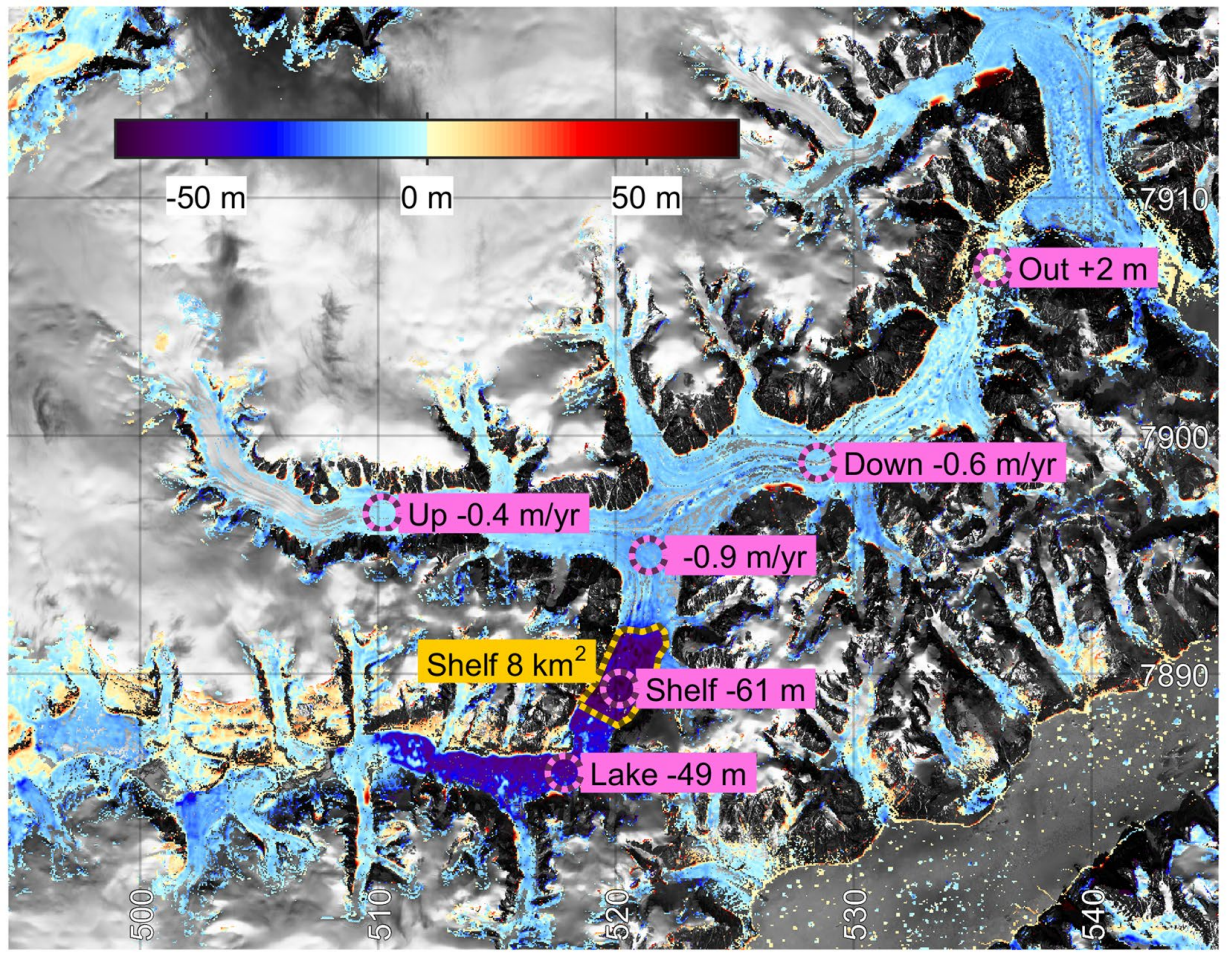
Elevation changes between 1986 and 2007 (AeroDEM to GIMP DEM). Maps were created using MATLAB^23^.

Only a few direct observations of the lake level exist over time. The existing elevation data from the area are: 1) Aero Digitial Elevation Model (AeroDEM^10^) (stereo photos from 1985/1987), 2) Greenland Mapping Project Digital Elevation Model (GIMP DEM^11, 12^) (data from 2007), 3) ICESat satellite laser altimetry data^8^ (2003–2009) along few tracks, and 4) Operation IceBridge^13^ (airborne ATM data along a flightline from 2014) (Supplementary Figure S2). The two DEMs and the IceBridge data agree over land, and clearly indicate the horizontal lake surface (Supplementary Figure S3). The data show different lake levels with the lowest level observed from the 2014 IceBridge data, thus providing an accurately measured low lake level and revealing part of the lake bottom. Comparison between the AeroDEM and the GIMP DEM show that Edward Bailey Glacier has thinned during the 10 year period from 1985/87 to 2007 by 0.4–0.6 m a^−1^ (Fig. 2). The lake level dropped 50 m between the two DEMs, and the glacier tongue terminating in Catalina Lake dropped by 60 m, clearly indicating that the glacier terminates in a floating tongue (Fig. 2). The area of the floating shelf is ~8 km^2^, thereby adding to the water volume draining from the lake. It is noted that the ice-free area downslope from the terminus of Edward Bailey Glacier increased by ~2 m between the two DEMS suggesting deposition of sediments (Fig. 2).

**Figure 3:**
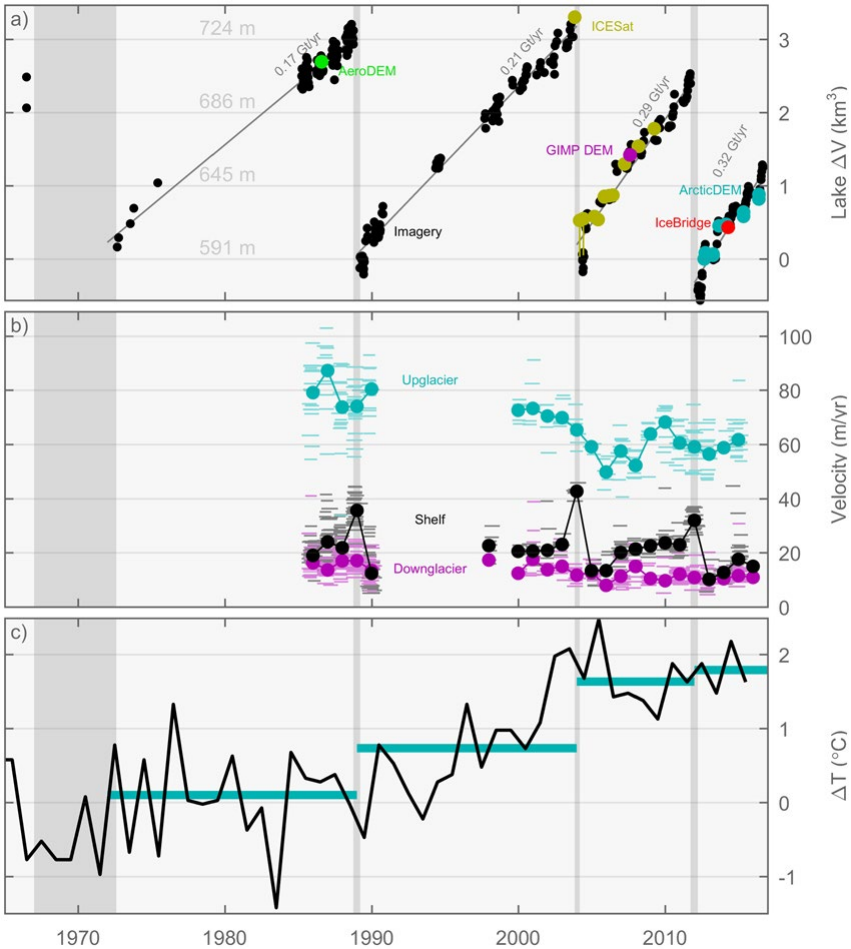
Satellite observed evolution of outburst floods and climate. (**a**) Catalina Lake volume and level estimated from shorelines(black), ICESat(yellow), AeroDEM(green), GIMP DEM(magenta), ArcticDEM(cyan), IceBridge(red). (**b**) Velocities at three locations on the glacier damming the lake estimated from optical feature tracking. Velocities for individual image pairs are shown as horizontal lines, and annual averages are shown as circles. (**c**) Regional temperatures relative to 1961–1990 (average of records from Danmarkshavn and Tassilaq). Cyan lines show longer term averages.

The long record of visible imagery from the area is used to reconstruct a record of lake level variations over time since the first available satellite images in 1966 (Fig. 3). We have developed a method to quantify the temporal variations in lake level from visible imagery by digitizing the lake shoreline on the satellite images and obtaining the elevation of the shoreline by matching the shoreline to elevation contours on the digital elevation model (DEM) of the area (See methods). We have no DEM of the lake bathymetry, however ArcticDEM provides us with data from shortly after a drainage event. We construct a lake bed DEM by interpolating between non-lake points in ArcticDEM, and the shoreline from 29-Aug-2012 which we assign to have a lake level of 591 m (as estimated from ArcticDEM 3 days later). Below this level, and below the shelf the lake bed is extrapolated from the surrounding landscape (See methods). Changes in lake level can now be determined from the lake bed DEM and related to changes in water volume (Supplementary Figure S4). We used all available images from Landsat 1–8, ASTER and declassified images^14^ to determine the lake level and the corresponding variations in water volume (See Supplementary dataset 1).

The resulting record of lake volume changes over time shows that outburst floods occurred four times within our observation time since the first available satellite images from 1966: between 1966–72, in winter 1988/89, in winter 2003/04, and in winter 2011/2012 (Fig. 3a). The timing of the first drainage cannot be accurately constrained due to lack of data, but the lower lake level in 1972 suggests a recent drainage of the lake. We observe a seasonal variation in filling rate, with the greatest rates in late July/early August. Our best constraints on the seasonal timing is from the 2003/04 ICESat data where we find it had not yet drained by Nov 10^th^. We speculate that the delay of several months between melting season and lake drainage is due to a yearly cycle in Edward Bailey glacier that is damming the lake, e.g. a winter slowdown of the ice flow into the shelf, and a thinning of the dam. The record shows consistent cycles of lake filling to a maximum threshold level at 734 m a.s.l. and drainage to a minimum level at 591 m a.s.l. (See Supplementary Table 1), corresponding a drainage of 3.3 ± 0.4 Gt (2σ; Supplementary Figure S4). The uncertainty in shoreline derived lake levels varies with image quality. The relative uncertainty in lake levels based on imagery since 1985 is estimated to be ± 7 m (2σ). We estimate the absolute uncertainty to be ± 8 m (2σ) by comparing shoreline levels to estimates from ICESat, AeroDEM, IceBridge, GIMP DEM, and ArcticDEM (see methods). The water volume filling rate increases slightly over each cycle, probably related to warmer climate in the region^15^ leading to increasing amounts of meltwater draining into the lake (Fig. 3c and Supplementary Figure S6). A slightly lower flood threshold level in the last outburst flood could be related to long-term thinning of the ice dam (Fig. 2). It has been previously proposed that an increasing rate of lake filling could lead to a higher drainage threshold^6^, but more data would be needed to understand the effect of filling rate on the flood threshold level for Catalina Lake.

Ice flow velocities of Edward Bailey glacier and the calving glacier branch were investigated to gain insights of the dynamics of the glacier dam (Fig. 3b). The annual mean surface velocities were determined from satellite images using a feature tracking method^16^ at three locations: at Edward Bailey Glacier upstream and downstream from the branch to Catalinadal valley, respectively, and at the floating glacier tongue in Catalina Lake (locations marked as ‘up’, ‘down’, and ‘shelf’ in Fig. 2. The flow of the Edward Bailey Glacier has a decelerating trend, particularly pronounced at the upstream location, and it does not respond directly to the drainage events. The deceleration may be related to the general thinning of the glacier of ~0.5 ma^−1^ observed between 1985/1987 and 2007 (Fig. 2). The flow velocity of the floating glacier tongue has a clear periodic response to the drainage cycle. The flow velocity peaks in the year of the drainage, then reaches a minimum value of about half the maximum velocity in the following year, and finally stabilizes on an intermediate velocity during the lake filling period. The acceleration coinciding with the flood may suggest that the glacier is lifted by flotation caused by the rising water level in the lake, thereby decreasing buttressing effects from the glacier bed and leading to an acceleration of the floating tongue. After the outburst flood, the lake level has lowered, and the deceleration of the floating tongue may suggest that the glacier becomes grounded again and flows slower while it recovers to a stable configuration as before the outburst flood.

The observations suggest that the outburst floods occur when lake level rises to the threshold point where the glacier dam can be floated by the lake water in accordance with the prevailing hypothesized causal mechanism of GLOFs^1, 3, 17^. As water begins to penetrate beneath the glacier, it starts to melt the glacier from below. The 6 km long glacier is branching out from Edward Bailey Glacier, and the lake water must overcome this obstacle and penetrate upstream the glacier flow before the drainage can occur under Edward Bailey Glacier. This configuration presumably explains the delay in the outburst flood from the summer melt season, where the main inflow of water to the lake is observed to occur. Information on ice thickness and bottom slopes are required to investigate this further. Observations from other Jökulhlaups find that floodwaters emerging at the glacier terminus are at the melting point, indicating that all available energy is spent on ice melt^18, 19^. The potential energy released during the outburst floods is in the order of 10^16^ Joules due to the 591–734 m high elevation of the lake, which would be sufficient to melt an additional water amount of 0.04 Gt, corresponding to a 20 km subglacial tunnel with a diameter of 55 m. A minimum estimate of discharge over the 2003/04 event is 200 m^3^s^−1^ found as an average discharge over the four month gap between observations. However, an empirical relation between peak discharge and drained lake volume^1, 20, 21^ suggest a peak discharge of 10^4^ m^3^s^-1^, about the maximum peak discharge rates observed in historical times^1^. The visible widening of the riverbed downstream from the terminus of Edward Bailey Glacier (Supplementary Figure S1), and the downstream deposition of ~2 m of sediments observed over an outburst flood (Fig. 2), support that the outburst flood drained through a subglacial tunnel with a peak water flux able to transport and redistribute significant amounts of sediments.

We can speculate when the next drainage event might occur by extrapolating the current rate of filling (Figure S6) to an assumed GLOF trigger level. The lake level would reach the level of the 2003–04 event by Jun 2023. However, continued thinning of the ice dam could allow the lake to drain sooner. E.g. a trigger level of 2 Gt correspond to an outburst in the winter 2018–19.

## Methods

In satellite imagery, we can visually track how the areal extent of Catalina Lake changes over time as it fills and drains (Fig. 1), as well as landscape changes in the outwash plain beneath Edward Bailey Glacier (Supplementary Figure S1).

We have developed a method to quantify the temporal changes in lake level by digitizing the lake shoreline on visual images and matching the shore outline to an elevation contour from a digital elevation model (DEM) of the area, thereby obtaining the elevation of the lake level.

We used all available images from Landsat 1–8, ASTER and declassified images from a CORONA photo intelligence satellite to identify the lake shoreline and track the lake level since the first available images from 1966 until 2016. The lake shoreline was digitized on the satellite images using QGIS software to provide the shoreline shape as a line polygon^22^. There are several sources of uncertainties in obtaining the lake level from these shoreline shapes: 1) the landscape characteristics combined with the image resolution, 2) georeferencing errors of the images, and 3) the absence of a complete DEM of the lake bottom.

Several DEMs are available for Renland (Supplementary Figure S2). The most recent DEM is ArcticDEM which is based on WorldView imagery from 2012–2016, when the lake level was low. Additionally, ArcticDEM provides incomplete snapshots during the same period (Supplementary Table 1). Another DEM is the Greenland Mapping Project Digital Elevation Model (GIMP DEM^11^). In Catalina Valley it is based on the SPIRIT DEM^12^, which was constructed from high-resolution SPOT-5 imagery acquired in 2007, when the lake was half-full. The last available DEM is the Aero Digitial Elevation Model (AeroDEM^10^), which is based on stereo photos from 1985/1987, when the lake level was high. Other available elevation data from Catalina Lake are obtained from the ICESat satellite laser altimetry data (2003–2009^8^) along few tracks crossing the lake, and from Operation IceBridge airborne ATM data^13^ along a flight line from 2014 (Supplementary Figure S2). These observations are listed in Supplementary Table 1.

Our method requires that we have an elevation model of the empty lake. ArcticDEM provides an elevation model from shortly after a drainage event. We used this as the basis for constructing a DEM of the lake bed. This was supplemented with the shoreline from 29-Aug-2012, which we assign to have a lake level of 591 m (as estimated from ArcticDEM 3 days later). Below this level the bed is extrapolated, and below the shelf, the lake bed is extrapolated from the surrounding landscape using Tikhonov regularization (Supplementary Figure S3).

Using the DEM of the lake bed, changes in lake level can now be explicitly related to changes in lake water volume. According to the DEM, a drop in lake level from 700 m to 600 m corresponds to a drainage of 2 Gt (Supplementary Figure S4). There is an uncertainty associated with lake geometry below the lake level in ArcticDEM. However, the greatest source of uncertainty is associated with the areal extent of the part of the lake that is covered by the floating ice tongue (Fig. 2). We assess the uncertainty of the lake level to volume calculation by generating a whole ensemble of alternative lake bathymetries using different options for the gridding (1^st^ vs 2^nd^ order Tikhonov regularization; different grid resolutions; varying smoothness constraints; varying shelf area).

The landscape of Renland is very dramatic, and Catalina Lake is flanked by steep valley slopes, with gradients that may exceed 50% in places. The steep slopes mean that even small horizontal errors translate to large vertical errors. The spatial resolution of the Landsat−1 scenes used in this study is ~60 m/pixel, and the most recent Landsat-8 has a resolution of 15 m/pixel. Uncertainties in the order of tens of meters are thus introduced. Another source of uncertainty is georeferencing errors in the satellite images. Older Landsat scenes occasionally have kilometer scale horizontal errors in the georeferencing. In new scenes, we observe considerable errors in the Landsat terrain correction within the study region. This manifests itself as large multi-pixel residual perspective offsets between Landsat scenes from different orbits. These georeferencing errors result in near uniform horizontal offsets in the lake shoreline. We determine this offset by aligning the shoreline with the geometry of the elevation model by minimizing the mean absolute deviation of the elevation along the shoreline (Supplementary Figure S5). We proceed to estimate the lake level as the median elevation along the shoreline to get a robust estimate of the lake level. We exclude certain small regions from contributing to the alignment procedure and from the level estimate. Excluded areas include glacier tongues meeting the lake, near vertical cliff faces, and areas where the local lake level consistently show disagreement with the remainder of the shoreline even after alignment. The method is useful even when part of the shoreline is invisible due to shadows, clouds or missing data.

Catalina Lake was imaged in 1966 by a CORONA photo intelligence satellite, and these data was declassified in 1996^14^. The CORONA images are of very high quality and resolution, but are more challenging to use, as they have not been georeferenced. We apply a first order georeferencing to the 1966 image by using common ground control points between 1966 and a recent Landsat-8 scene. The ground control points were chosen in the neighborhood of the lake. We transform the image using both an affine and a spline transform, and trace the shoreline in both. The lake level is estimated following a similar approach to the Landsat derived shorelines, but we allow for a full affine correction when we align the shoreline to the elevation model.

### Data availability

All data used in this study are available from open archives (see references in manuscript). Data generated during this study are included in this published article (and its Supplementary Information files).

## Electronic supplementary material

Supplementary information

Supplementary Dataset
