# Supplementary material for: Periodic outburst floods from an ice-dammed lake in East Greenland
Source: Sci Rep. 2017 Aug 30;7:9966. doi: 10.1038/s41598-017-07960-9 (PMC5577127; doi:10.1038/s41598-017-07960-9)
Supplement: Supplementary file 1 — Supplementary information [file 41598_2017_7960_MOESM1_ESM.pdf]

Title: Periodic outburst floods from an ice-dammed lake in East Greenland.

Authors: Aslak Grinsted[1]*, Christine S. Hvidberg[1], Néstor Campos[2], Dorthe Dahl-Jensen[1].

Affiliations:

[1] Centre for Ice and Climate, Niels Bohr Institute, University of Copenhagen, Juliane Maries Vej 30, DK-2100 Copenhagen, Denmark.

[2] Research Group of High Mountain Physical Geography, Complutense University of Madrid, 28040 Madrid, Spain.

*Correspondence to: ag@glaciology.net

Supplementary Material:

Supplementary figures S1-S6.

Supplementary table 1

Supplementary dataset 1 *in separate excel file.

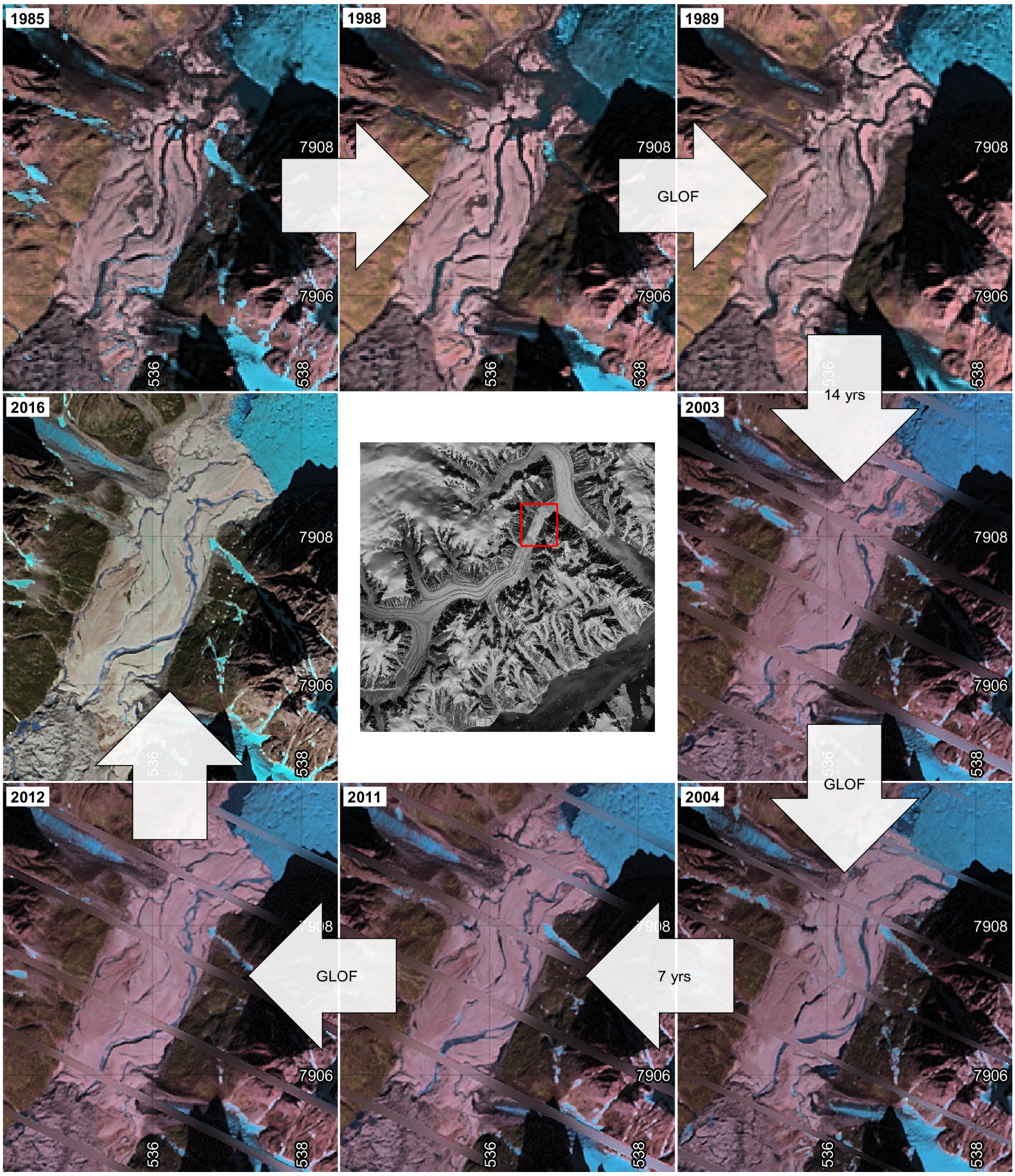

Figure S1: Landscape change in front of Edward Bailey Glacier. Landsat summer images show landscape changes in the outwash plain below Edward Bailey Glacier between 1985 and 2015 covering three glacier lake outburst floods (GLOFs). The images are ordered in a clockwise orientation as indicated by arrows. During GLOFs the landscape downstream from the terminus changes. No discernable changes occurred between GLOFs. Maps were created using MATLAB (2016).

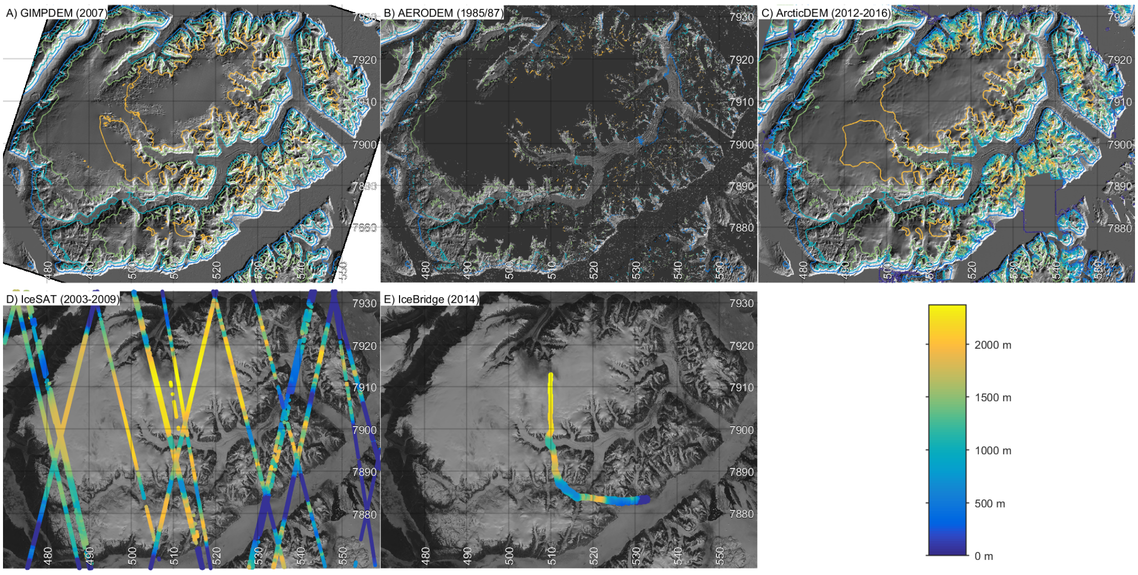

Figure S2: Elevation data used in this study. A) AeroDEM 1985/1987; B) GIMP DEM 2007; C) ArcticDEM (2012-2016); D) ICESat (2003-2009); and E) IceBridge ATM data from 2014. Maps were created using MATLAB (2016).

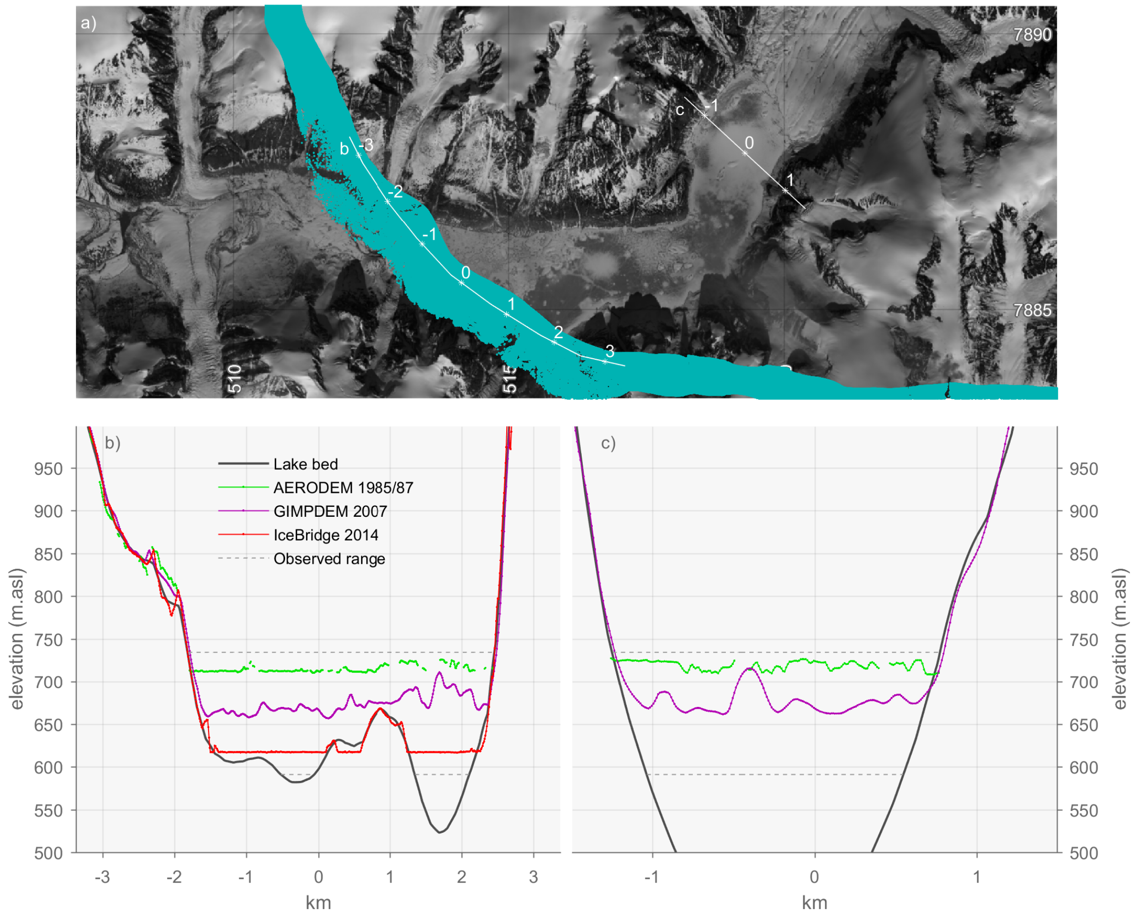

Figure S3: a) Map of catalinadal with IceBridge ATM data(cyan), and locations of slices in white. b) and c) slices through the elevation models at the two locations. The bump and upsloping level towards the northern lakeshore in the IceBridge lake surface data from 2014 are real, showing the lake bottom gradually being revealed at low water level. Map was created using MATLAB (2016).

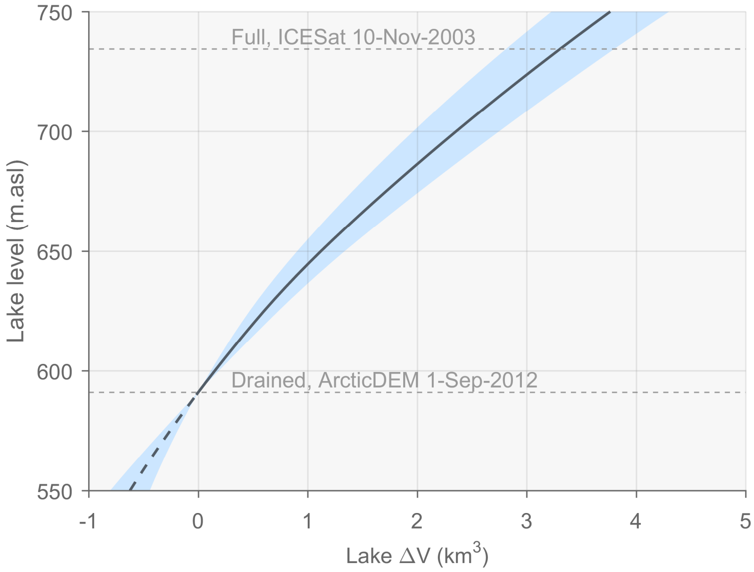

Figure S4: Relationship between lake volume and lake level. The relationship is estimated from the lake bed DEM as described in the text. The shaded region shows a 2σ uncertainty interval.

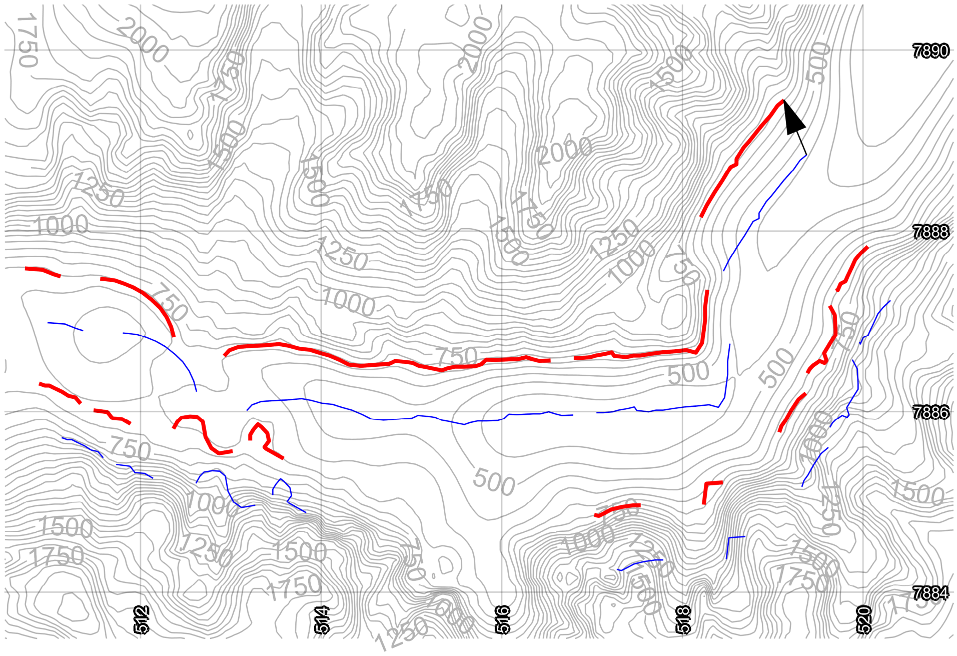

Figure S5: Illustration of the method. The digitized shoreline (blue) is matched (red) to an elevation model (contour lines) by minimizing the elevation deviation along the shoreline, and the lake level is inferred. Maps were created using MATLAB (2016).

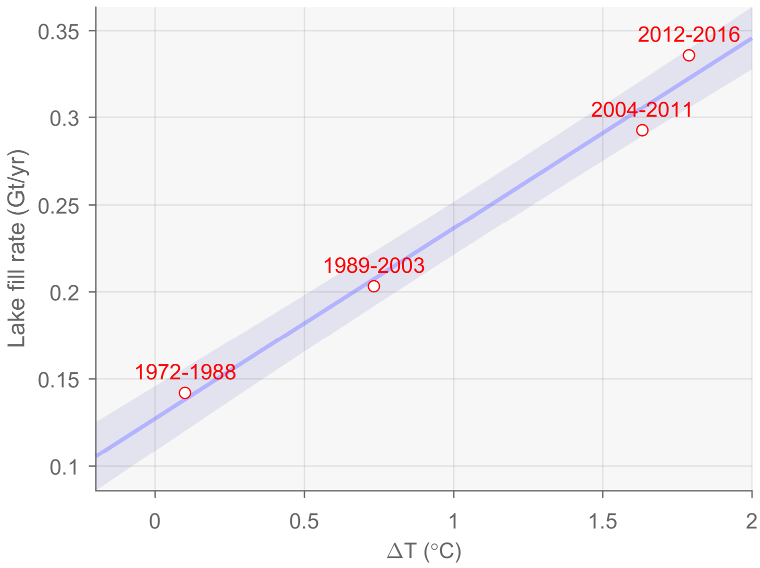

Figure S6: Relationship between the lake water volume filling rate and the regional temperature. Warmer regional temperatures has been associated with a faster filling of the lake between GLOF events. Shading shows the 2σ uncertainty of the regression line (blue). Temperatures are calculated as the average of the temperatures at Danmarkshavn and Tassilaq relative to a 1961-1990 baseline.

Supplementary table 1: Lake level observations (Z) from ICESat, IceBridge, AeroDEM, GIMP DEM, and ArcticDEM. The levels are compared to shoreline derived lake levels ($Z_{shoreline}$). *In 2004 ICESat went over a freshly exposed part of the lake bed and can therefore only serve as an upper bound on the lake level.

Source	Date	Z	$Z - Z_{shoreline}$
ICESat	10-11-2003	734 m	9.5 m
ICESat	13-03-2004	621 m*	26.2 m*
ICESat	12-06-2004	622 m*	38.8 m*
ICESat	16-03-2005	624 m	0.2 m
ICESat	15-06-2005	622 m	-1.1 m
ICESat	15-11-2005	637 m	4.2 m
ICESat	23-11-2005	638 m	4.5 m
ICESat	19-03-2006	638 m	2.7 m
ICESat	18-06-2006	638 m	2.0 m
ICESat	06-04-2007	658 m	-1.0 m
ICESat	13-03-2008	668 m	4.0 m
ICESat	03-04-2009	678 m	6.1 m
IceBridge	21-04-2014	616 m	-2.3 m
AeroDEM	05-08-1986	713 m	5.6 m
GIMP DEM	18-08-2007	663 m	3.1 m
ArcticDEM	01-09-2012	591 m	-5.6 m
ArcticDEM	27-09-2012	596 m	-6.8 m
ArcticDEM	09-04-2013	595 m	4.8 m
ArcticDEM	10-04-2013	594 m	3.8 m
ArcticDEM	06-09-2013	617 m	-3.0 m
ArcticDEM	19-02-2014	618 m	-2.0 m
ArcticDEM	24-02-2014	617 m	-3.0 m
ArcticDEM	04-03-2014	618 m	-0.6 m
ArcticDEM	09-05-2015	624 m	-4.2 m
ArcticDEM	10-05-2015	627 m	-1.2 m
ArcticDEM	24-05-2016	636 m	0.8 m
ArcticDEM	27-05-2016	639 m	-0.2 m

Acknowledgments.

The research was funded by ERC Advanced Grant no 246815 (WATERundertheICE) and Villum Investigator Project IceFlow. Landsat 1-8, ASTER, and declassified imagery are distributed by the Land Processes Distributed Active Archive Center (LP DAAC), located at USGS/EROS, Sioux Falls, SD. http://lpdaac.usgs.gov. We used ArcticDEM data, created from DigitalGlobe, Inc., imagery 2012-2016, and provided by the Polar Geospatial Center under NSF OPP awards 1043681, 1559691 and 1542736.
